# Optimization of 4D combined angiography and perfusion using radial imaging and arterial spin labeling

**DOI:** 10.1002/mrm.29558

**Published:** 2022-12-19

**Authors:** Thomas W. Okell, Mark Chiew

**Affiliations:** ^1^ Wellcome Centre for Integrative Neuroimaging, FMRIB, Nuffield Department of Clinical Neurosciences University of Oxford Oxford UK

**Keywords:** 3D radial MRI, arterial spin labeling, dynamic angiography, non‐contrast, perfusion imaging, simultaneous acquisition

## Abstract

**Purpose:**

To extend and optimize a non‐contrast MRI technique to obtain whole head 4D (time‐resolved 3D) qualitative angiographic and perfusion images from a single scan.

**Methods:**

4D combined angiography and perfusion using radial imaging and arterial spin labeling (CAPRIA) uses pseudocontinuous labeling with a 3D golden ratio (“koosh ball”) readout to continuously image the blood water as it travels through the arterial system and exchanges into the tissue. High spatial/temporal resolution angiograms and low spatial/temporal resolution perfusion images can be flexibly reconstructed from the same raw k‐space data. Constant and variable flip angle (CFA and VFA, respectively) excitation schedules were optimized through simulations and tested in healthy volunteers. A conventional sensitivity encoding (SENSE) reconstruction was compared against a locally low rank (LLR) reconstruction, which leverages spatiotemporal correlations. Comparison was also made with time‐matched time‐of‐flight angiography and multi‐delay EPI perfusion images. Differences in image quality were assessed through split‐scan repeatability.

**Results:**

The optimized VFA schedule (2–9°) resulted in a significant (*p* < 0.001) improvement in image quality (up to 84% vs. CFA), particularly for the lower SNR perfusion images. The LLR reconstruction provided effective denoising without biasing the signal timecourses, significantly improving angiographic and perfusion image quality and repeatability (up to 143%, *p* < 0.001). 4D CAPRIA performed well compared with time‐of‐flight angiography and had better perfusion signal repeatability than the EPI‐based approach (*p* < 0.001).

**Conclusion:**

4D CAPRIA optimized using a VFA schedule and LLR reconstruction can yield high quality whole head 4D angiograms and perfusion images from a single scan.

## Abstract 2

Click here for author‐reader discussions

## 1 INTRODUCTION

The ability to visualize blood flow to the brain, both through the arterial system (angiography) and at the level of the tissue (perfusion), is crucial in many cerebrovascular diseases, including stroke and arteriovenous malformation.^1^ X‐Ray–based methods require the administration of an exogenous contrast agent and exposure to ionizing radiation. Contrast‐enhanced MRI methods avoid ionizing radiation, but often have limited spatiotemporal resolution due to the necessity to image the first passage of the bolus,^2^ and there are concerns about the use of gadolinium‐based contrast agents in patients with kidney disease,^3^ as well as accumulation in the brain.^4^ A non‐contrast alternative is therefore desirable.

Arterial spin labeling (ASL) is an MRI‐based non‐contrast method capable of generating both angiograms^5^, ^6^, ^7^ and maps of tissue perfusion.^8^, ^9^ However, the high spatial and temporal resolution required for angiography makes time‐resolved 3D ASL angiograms slow to acquire in a conventional manner,^2^ whereas ASL perfusion images require the acquisition of many averages to improve the SNR.^10^ Therefore, obtaining both angiographic and perfusion information using ASL within a busy clinical protocol is often challenging.

Recently, a few methods have been proposed to address this problem by acquiring angiograms and perfusion maps simultaneously from a single ASL acquisition, thereby increasing the time‐efficiency. This has involved either the use of two separate readout modules for angiography and perfusion imaging,^11^ or the use of a single golden ratio^12^ radial readout.^13^, ^14^ This latter approach allows flexibility in the spatial and temporal resolution of the reconstructed images, thereby allowing high spatial/temporal resolution angiograms and lower spatial/temporal resolution perfusion maps to be generated at multiple time points after the labeling period from the same raw k‐space data. However, this approach has thus far only been demonstrated using single or multi‐slice 2D readouts, limiting the spatial coverage and SNR achievable.

In this work, we extend and optimize the image quality of one of these methods, Combined Angiography and Perfusion using Radial Imaging and ASL (CAPRIA),^13^ by: (1) utilizing multi‐dimensional golden means to allow fully isotropic 4D (time‐resolved 3D) imaging of the whole head; (2) modulating the ASL signal using a variable flip angle (VFA) readout, mitigating signal attenuation due to the imaging pulses; and (3) applying an advanced image reconstruction technique to leverage spatiotemporal signal correlations and minimize noise propagation in these undersampled acquisitions. Here we focus on optimizing qualitative angiography and perfusion imaging. The extraction of quantitative physiological parameters from this kind of data will be explored in future work. This study builds upon work previously presented in abstract form.^15^, ^16^, ^17^

## 2 METHODS

### 2.1 Pulse sequence design

**FIGURE 1:**
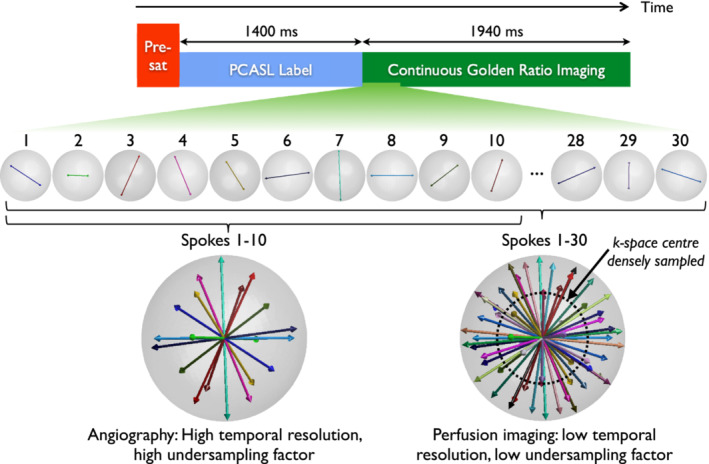
4D CAPRIA sequence schematic. A WET pre‐saturation module (pre‐sat) is followed by PCASL labeling and then continuous 3D golden ratio imaging, providing flexibility for the image reconstruction. In this simple example, sets of 10 spokes can be used to reconstruct angiographic images with a high temporal resolution (small temporal window) at any time point within the readout period. This results in a high undersampling factor, but this can be tolerated for the sparse, high SNR angiographic signal. The same raw k‐space data can be reconstructed at a lower temporal resolution (larger temporal window, 30 spokes used here), which is better suited to the slower dynamics of perfusion imaging. This results in a lower undersampling factor, necessary for the low SNR and less sparse perfusion signal. Using just the densely sampled center of k‐space, lower spatial resolution perfusion images can be reconstructed, further reducing the undersampling factor

A schematic of the 4D CAPRIA sequence is shown in Figure 1. The preparation period is identical to the original approach,^13^ consisting of a slab‐selective water suppression enhanced through T_1_ effects (WET) pre‐saturation module^18^ for background suppression and a 1400 ms duration balanced pseudocontinuous (PCASL)^19^ pulse train to label blood flowing through a plane in the neck (positioned in a similar manner to previous studies,^20^, ^21^ through the distal V2 segment of the vertebral arteries, ˜1 cm below the start of the V3 segment).

This is followed by a continuous 3D golden ratio radial (“koosh ball”) spoiled gradient echo readout, where a single line (“spoke”) of data going through the k‐space origin is acquired following each of a series of non‐selective excitation pulses. The polar and azimuthal angles of each spoke are calculated according to the multi‐dimensional golden means,^22^ which is a 3D generalization of the original golden ratio approach.^12^ The azimuthal (*φ*) and polar (*θ*) angles of the *m* ^th^ radial spoke are:

(1) $$\theta =\cos^{-1}\left\{m\phi_{1}\right\}$$

(2) $$\varphi =2\pi \left\{m\phi_{2}\right\}$$

where *ϕ*_1_ = 0.4656…, *ϕ*_2_ = 0.6823… and the curly braces {.} denote taking the fractional part (i.e., modulus 1). This approach retains the same appealing property that any set of contiguously acquired spokes gives an approximately uniform coverage of k‐space, meaning that the temporal window used for image reconstruction (and therefore the temporal resolution, undersampling factor and post‐labeling delay [PLD]) can be arbitrarily chosen retrospectively.

As illustrated in Figure 1, the golden ratio trajectory allows angiographic images to be reconstructed with a small temporal window (high temporal resolution), which is required to capture the rapid flow of blood through the arterial system. This results in a high undersampling factor, but the spatially sparse and high SNR nature of the angiographic signal mean this can be tolerated.^13^, ^23^, ^24^ Due to T_1_ decay and the dilution of the labeled blood, the ASL signal is considerably weaker by the time it reaches the tissue. However, the dynamics of this perfusion signal are also slower, so a broader temporal window can be used to reconstruct perfusion images from the same raw k‐space data. In addition, the center of k‐space is much more densely sampled than the periphery, so lower spatial resolution images, as are typically used for perfusion imaging, can be reconstructed with a much lower undersampling factor. The ability to reconstruct angiographic or perfusion‐like images at any timepoint within the readout period retrospectively gives a great degree of flexibility to adapt to the hemodynamics of any individual subject.

In practice, data combined across many ASL preparation periods are required to achieve sufficient sampling. As previously,^13^ we define a maximum temporal window, *t*_max_, at which data will be reconstructed, which corresponds to *M* radial spokes (*M = t*_max_/TR). The golden ratio spoke counter, *m*, for the *i* ^th^ spoke acquired after the *n* ^th^ ASL preparation is then:

(3) m=i−1+(n−1)M.

In order to minimize subtraction artifacts due to factors such as scanner drift or motion the acquisition was interleaved such that the time between the same k‐space spokes being acquired for the ASL label and control conditions was as short as possible.

A readout partial Fourier (asymmetric echo) factor of 0.79 was used in order to reduce the TE and minimize flow‐induced dephasing effects. However, Eq. (1) results in each spoke starting on the surface of one hemisphere (0 < *θ* < π/2), so readout partial Fourier would lead asymmetric sampling of k‐space. Therefore, the direction of every other spoke was reversed to give a more even distribution, i.e., for odd values of *m*:

(4) $$\theta_{\mathrm{odd}}=\cos^{-1}\left(-\left\{m\phi_{1}\right\}\right)$$

(5) $$\varphi_{\mathrm{odd}}=2\pi \left\{m\phi_{2}+1/2\right\}.$$

Finally, two possible flip angle schedules for the excitation pulses were investigated: (a) a constant flip angle (CFA) approach and (b) a VFA approach, in which the flip angle of the *i* ^th^ pulse in the readout, *α*_*i*_, increases quadratically from *α*_1_ at the first pulse to *α*_*N*_ at the final (*N* ^th^) pulse as follows^25^:

(6) $$\alpha_{i}=\alpha_{1}+\left(\alpha_{N}-\alpha_{1}\right){\left(\frac{i-1}{N-1}\right)}^{2}.$$

This could help to minimize signal attenuation at early timepoints while boosting the signal at later timepoints, which is anticipated to be particularly beneficial for the CAPRIA perfusion signal that relies on the accumulation of labeled blood water over time.

### 2.2 Parameter optimization simulations

It was necessary to reoptimize the readout parameters (the TR of the excitation pulses and their flip angles, *α*_*i*_) for 4D CAPRIA in order to balance the need for fast acquisitions, low undersampling factors and strong initial signal against signal attenuation effects. Based on the parameters required for in vivo scanning (Table 1), a TR of 9 ms was chosen to minimize signal attenuation while keeping the undersampling factors within reasonable limits.

**TABLE 1:** Imaging and reconstruction parameters

			Conventional methods	
Parameter	CAPRIA		TOF	Multi‐PLD EPI
Label				
Labeling duration (ms)	1400		–	1400
Background suppression	Pre‐saturation		–	Pre‐saturation + two inversion pulses
Readout			
Acquired matrix size	160 isotropic		214 × 256 × 172	64 × 64 × 35
Nominal voxel size (mm^3^)	1.1 × 1.1 × 1.1		0.9 × 0.9 × 2.2	3.4 × 3.4 × 3.4
Nominal FOV (mm)	183 × 183 × 183		220 × 184 × 189	220 × 220 × 119
Flip angle (°)	6 (CFA) or 2–9 (VFA)		18	90
TE (ms)	3.4		3.6	14
TR (ms)	9.0		22	5002
Partial Fourier factor	0.79 (readout)		0.875 (slice)	0.75 (phase)
Bandwidth (Hz/Pixel)	99		186	2004
Imaging time after labeling (ms)	1939.7		–	1719.1 (49.1 ms per slice)
Max. temporal window, *t*_max_ (ms)	323.3		–	–
Spokes/lines per arterial spin labeling preparation, *N*	216		–	–
Label/control pairs	88		–	30 (5 per PLD)
Acquisition time (min:s)	9:59		4:52	5:00

	Angiography	Perfusion		
Reconstruction				
Temporal resolution (ms)	215.5	323.3	–	323.0
Time frames	9	6	–	6
Spatial resolution (mm^3^)	1.1 × 1.1 × 1.1	3.5 × 3.5 × 3.5	0.5 × 0.5 × 1.1 (interpolated)	3.4 × 3.4 × 3.4
Reconstructed FOV (mm)	194 × 263 × 284	194 × 264 × 285	220 × 184 × 189	220 × 220 × 119
Total spokes/lines per frame	2112	3168	–	–
Undersampling factor	19.0	1.3	2 (GRAPPA)	–
SENSE regularization factor	10^5^	10^5^	–	–
SENSE iterations	100	100	–	–
SENSE reconstruction time (h)	24	0.5	–	–
LLR regularization factor	10^−1^	7 × 10^−2^	–	–
LLR patch size (voxels × timepoints)	15 × 15 × 15 × 9	5 × 5 × 5 × 6	–	–
LLR iterations	200	200	–	–
LLR reconstruction time (h)	35	0.7	–	–

CAPRIA acquisition parameters were optimized by simulating the relative strength of the angiographic and perfusion signals across a range of possible parameter choices.^13^ The angiographic signal was evaluated using a kinetic model for PCASL angiography,^26^ assuming the labeled blood experiences all of the RF excitation pulses due to their non‐selective nature. However, the RF attenuation factor, *R*_*i*_, applied to the signal acquired after the *i* ^th^ RF pulse must be generalized to account for a VFA schedule:

(7) $$R_{i}=\left\{\begin{array}{ll} 1 & i=1\\ \prod_{j=1}^{i-1}\cos\alpha_{j} & i>1 \end{array}\right.$$

In addition, the VFA effect on the magnitude of the excited transverse magnetization must be also be accounted for. For this study, we also ignore the effects of dispersion to give a final simplified angiographic model:

(8) $$S_{\text{angio},i}=\left\{\begin{array}{ll} \;2M_{0b}\alpha_{\mathrm{inv}}\;v\;\sin\alpha_{i}exp(‐\delta_{t}/T_{1b})R_{i} & \delta_{t}<t_{i}\leq \delta_{t}+\tau \\ 0 & \text{otherwise} \end{array}\right.$$

where *t*_*i*_ is the time of excitation pulse *i* relative to the start of PCASL labeling, *δ*_*t*_ is the macrovascular transit time, *τ* is the PCASL labeling duration, *v* is the fractional macrovascular blood volume and we have expressed the scaling factor using the equilibrium magnetization of blood, *M*_*0b*_, and PCASL inversion efficiency, *α*_inv_.

Similarly, the CAPRIA perfusion signal,^13^ *S*_perf,*i*_, after the *i* ^th^ excitation pulse, must also be modified:

(9) $$S_{\text{perf},i}=\Delta M_{B,i}R_{i}\sin\alpha_{i}$$

where $\Delta M_{B,i}$ is the Buxton model for the (P)CASL perfusion signal.^27^

The angiographic and perfusion signals were simulated for a range of flip angle schedules (*α*_1_ and *α*_*N*_) across a range of physiological parameters. Since blood volume and blood flow (to a first approximation) only scale the angiographic and perfusion signals, respectively, it is not necessary to optimize over these parameters explicitly,^28^ leaving only the macrovascular transit time, *δ*_*t*_, and the tissue transit time, Δ*t*, to optimize over. Here, we optimized for 0.2 s < *δ*_*t*_ < 1 s and 0.5 s < Δ*t* < 2 s, based on typical values observed in previous healthy volunteer studies.^26^, ^28^

To compare different acquisition parameters, the mean angiographic signal was calculated during the time that labeled blood was present (i.e., *δ*_*t*_ ≤ *t*_*i*_ < *τ* + *δ*_*t*_). Similarly, the mean perfusion signal was calculated after all blood water had reached the voxel (*t*_*i*_ ≥ *τ* + Δ*t*) when the perfusion signal is approximately proportional to cerebral blood flow.^27^ These mean signals were then averaged across all physiological parameters and normalized to the maximum value to give a scalar performance metric for angiography, ${\bar{S}}_{\text{angio}}\left(\alpha_{1},\alpha_{N}\right)$ and perfusion, ${\bar{S}}_{\text{perf}}\left(\alpha_{1},\alpha_{N}\right)$. A combined measure, ${\bar{S}}_{\text{combined}}\left(\alpha_{1},\alpha_{N}\right)$, was also generated by a weighted sum of these two and renormalized:

(10) $${\bar{S}}_{\text{combined}}\left(\alpha_{1},\alpha_{N}\right)=\frac{1}{2}{\bar{S}}_{\text{angio}}\left(\alpha_{1},\alpha_{N}\right)+{\bar{S}}_{\text{perf}}\left(\alpha_{1},\alpha_{N}\right).$$

The arbitrary weighting factor of ½ was chosen on the assumption that the perfusion signal is considerably lower SNR and, therefore, should carry a higher weight.

### 2.3 Subjects and scan protocol

To demonstrate 4D CAPRIA and compare CFA and VFA approaches, four healthy volunteers (two female, age range 26–39) were scanned under a technical development protocol agreed by local ethics and institutional committees on a 3T Verio scanner (Siemens Healthineers, Erlangen, Germany) using a 32‐channel head coil. A short (29 s) time‐of‐flight (TOF) angiogram was acquired to help position the ASL labeling plane,^21^ along with a short (2.5 min) T1‐weighted structural image for anatomical reference (MP‐RAGE,^29^ 1.8 mm voxel size, 900 ms TI).

1.1 mm isotropic CFA and VFA 4D CAPRIA scans covering the head and upper neck were then performed using optimal flip angles derived from simulations (CFA: *α* = 6°; VFA: *α*_1_ = 2°, *α*_*N*_ = 9°). Other acquisition and reconstruction parameters are listed in Table 1.

### 2.4 CAPRIA image reconstruction

From each raw CAPRIA k‐space dataset, two sets of images were reconstructed: (1) angiographic images with high spatial/temporal resolution (1.1 mm isotropic, 216 ms, undersampling factor = 19.0); and (2) perfusion images with reduced spatial/temporal resolution (3.5 mm isotropic, 323 ms, undersampling factor = 1.3) to improve the SNR. To minimize computational requirements, perfusion images were reconstructed directly at a lower matrix size using data from the central region of k‐space.

Coil sensitivities were estimated by combining data across all time frames, averaging the label/control data and applying a Hann filter to minimize signal aliasing arising from undersampling. The non‐uniform fast Fourier transform (NUFFT)^30^, ^31^ operator was then used with a short conjugate gradient iterative reconstruction to produce one image per coil element at the same resolution intended for reconstruction prior to coil sensitivity estimation^32^ and masking. Coil compression from 32 to 12 channels was performed to reduce computational burden.^33^

The phase of the same k‐space spokes acquired in label and control conditions were aligned^34^ prior to complex subtraction in k‐space to minimize any phase inconsistencies. The ASL difference images were then reconstructed directly using two different approaches: (1) conventional iterative conjugate gradient SENSE^35^ with L2 regularization on spatial and temporal gradients; and (2) a locally low rank (LLR) approach,^36^ using cycle spinning^37^ and the proximal optimized gradient method,^38^ to leverage spatiotemporal correlations in the patterns of blood flow.

All reconstruction stages were run using MATLAB 2017b (Mathworks, Natick, MA), incorporating the IRT NUFFT,^31^ on a CentOS cluster. Regularization factors and LLR patch sizes were chosen empirically based on preliminary experiments (see Table 1). The absolute value of the complex angiographic and perfusion signals was taken prior to visualization and further analysis.

### 2.5 Repeatability

Conventional SNR measurements are challenging in this setting due to signal aliasing, spatially varying noise and the use of a non‐linear reconstruction method (LLR). Therefore, as an alternative measure of image quality, a repeatability metric was calculated to give an indication of signal stability. The raw k‐space data was split into two halves, each half used to reconstruct angiograms and perfusion images, and the Pearson correlation coefficient between the two halves calculated at each timepoint within a spatial mask. This mask excluded large regions of noise‐only background which could bias the correlation metric.

For perfusion imaging, a whole brain mask was used, generated from the T1‐weighted structural image (using fsl_anat^39^) and linearly registered^40^ to the CAPRIA data. For angiography, a dilated vessel mask was created to include all the major vessels and some background by: averaging CFA and VFA angiograms reconstructed with SENSE, taking the temporal mean, multiplying by the brain mask, thresholding at 0.7 × 99.9^th^ percentile image intensity, then dilating this mask using a spherical kernel (radius 3.5 mm). An example mask is shown in Figure S1.

Prior to performing statistical analyses, these correlation coefficients, *r*, were Fisher transformed^41^ as follows:

(11) $$z=\tanh^{-1}r.$$

A multi‐way ANOVA was performed to assess the statistical significance of differences in *z* between CFA and VFA schedules or SENSE and LLR reconstructions, with subject number and PLD as additional factors.

### 2.6 Comparison with conventional methods

In three of the four subjects, angiograms and perfusion images were also acquired using conventional approaches with the total scan time matched to that of CAPRIA (˜5 min each). Conventional angiography was a 3D multi‐slab TOF protocol. Due to restrictions within the sequence protocol and the requirement to match the spatial coverage of the CAPRIA data within a 5 min scan, it was not possible to exactly match the CAPRIA spatial resolution, despite using in‐plane parallel imaging acceleration (GRAPPA factor 2) with 7/8th partial Fourier in the slice direction. Slice resolution had to be compromised, resulting in a voxel size of 0.9 × 0.9 × 2.2 mm^3^. Multi‐PLD PCASL perfusion images were also acquired using a 2D multi‐slice EPI readout, similar to a previous study,^21^ with nominal postlabeling delays to closely match the reconstructed CAPRIA perfusion images (185, 485, 808, 1131, 1454, and 1777 ms) and comparable spatial resolution (3.4 × 3.4 × 3.4 mm^3^). However, note that the time to acquire each slice (49.1 ms) results in longer effective PLDs in more distal slices. There was sufficient time for five label‐control pairs to be acquired at each PLD. More detailed parameters for both protocols are given in Table 1.

Both sets of conventional images were reconstructed using the vendor‐provided online reconstruction algorithms. PCASL EPI perfusion images were motion corrected with MCFLIRT^40^ before control‐label subtraction and averaging across repeats at each PLD. It was not possible to retrospectively split the TOF data to assess split‐scan repeatability, so only qualitative comparisons with CAPRIA were performed. For the perfusion data, an approximate split scan repeatability metric was calculated. Since there was only time for 5 label‐control pairs at each PLD, the final pair was discarded before averaging data from pairs 1 and 2 and correlating that with averaged data from pairs 3 and 4. This will increase the noise in each “half” of the data, reducing the apparent repeatability metric. This was corrected for by simulating the correlation coefficients calculated from a range of signals across different noise levels (*r*_full_) and comparing those to the correlation coefficients which would be obtained if the scan time were reduced by a factor of 4/5, resulting in $\sqrt{5/4}$ higher noise level (*r*_reduced_). This resulted in the following empirical correction, which was applied to the correlation coefficients derived from the first four label‐control pairs of EPI perfusion data:

(12) $$r_{\text{full}}=-0.2231r_{\text{reduced}}^{2}+1.2221r_{\text{reduced}}+0.0004.$$

### 2.7 Potential for scan time reduction

The full 4D CAPRIA protocol described above took 10 min, which may be too long to fit within a busy clinical protocol. In order to demonstrate the potential for a more rapid, clinically relevant scan, angiograms and perfusion images reconstructed using LLR from the full 10 min of data were qualitatively compared with those reconstructed from only the first half of the data (5 min). Note that, due to the golden ratio ordering scheme used, this is exactly equivalent to a shorter prospective scan, since it is only data acquired after the first 5 min which is discarded before reconstruction is performed.

## 3 RESULTS

### 3.1 Parameter optimization simulations

**FIGURE 2:**
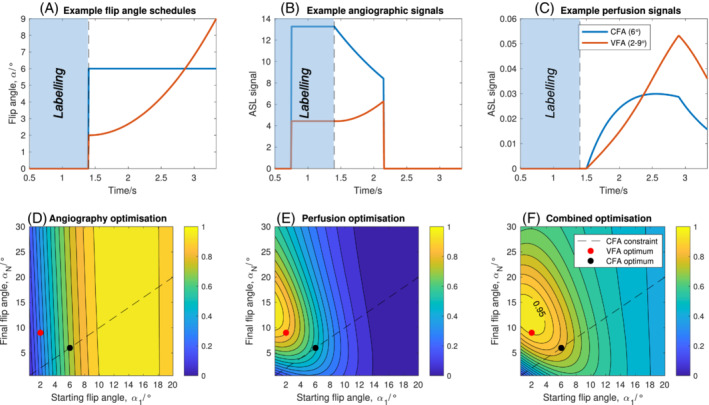
Flip angle schedules (A), simulated angiographic signals (B), and simulated perfusion signals (C) for one example constant flip angle schedule (CFA = 6°) and one quadratically varying flip angle schedule (VFA = 2–9°), demonstrating the potential for considerable signal gains in the perfusion images using VFA (these plots assume transit times of 0.75 s and 1.5 s to the arterial and perfused tissue voxels, respectively). Optimization of the flip angle parameters to maximize signal strength over a range of physiological parameters is shown for angiography (D), perfusion (E), and a combination (weighted sum) of the two (F). Each contour plot is normalized, the constraint corresponding to a constant flip angle shown as a dashed line, and the final chosen optimum CFA and VFA parameters plotted as black and red circles, respectively

Example CFA and VFA schedules and the resulting simulated angiographic/perfusion signals are shown in Figure 2A–C. As expected, the use of a CFA schedule results in the attenuation of both the angiographic and perfusion signals over time, but it is particularly problematic in the perfusion case due to the later blood arrival times. In contrast, the VFA schedule results in a smaller, but more consistent, angiographic signal that increases slightly over time before the labeled blood water washes out. However, it has a double benefit for the perfusion signal: reduced attenuation at early timepoints means a larger perfusion signal can accumulate within the voxel, but also the amount of signal generated by the excitation pulses at later timepoints is higher. In the case shown (Δ*t* = 1.5 s), this resulted in a 2.3× larger perfusion signal at the end of the readout.

The results of optimizing the flip angle schedules across a range of physiological parameters are shown in Figure 2D–F. The angiographic optimization favors larger starting flip angles with less dependence on the final flip angle, since most of the signal is present at early timepoints. Conversely, the perfusion optimization favors low starting flip angles and higher final flip angles. The combined optimization metric had a broad peak, so the (*α*_1_, *α*_N_) combination that was within 95% of the peak value but closest to the origin was chosen for the remainder of the study (2–9°) to minimize the signal attenuation effect on late arriving blood. The CFA schedule is constrained to lie on the line *α*_1_ *= α*_*N*_, which gives considerably poorer predicted performance for the perfusion and combined optimization metrics than the VFA approach. The CFA which optimized the combined metric was 6° and this was used for the remainder of this study.

### 3.2 In vivo results

**FIGURE 3:**
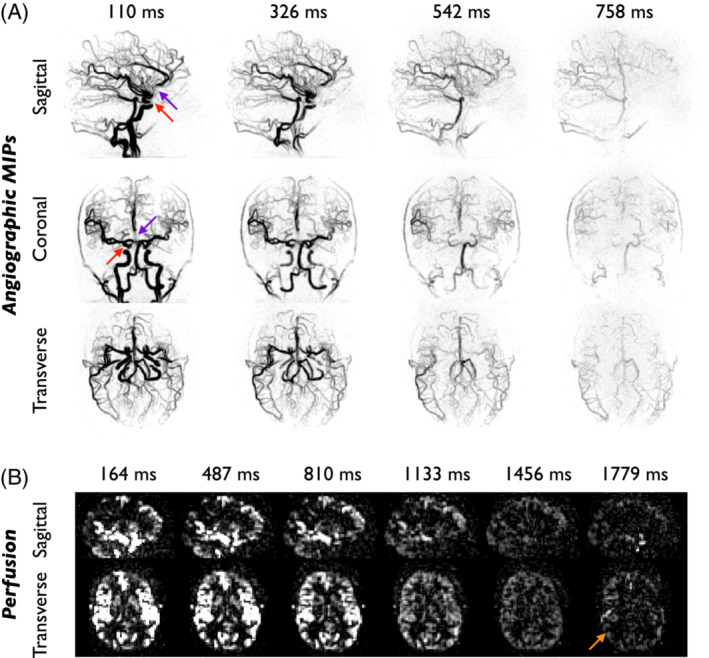
Example CAPRIA SENSE reconstructions in subject 1 using a constant flip angle (CFA) schedule. (A) Angiography maximum intensity projections in inverted greyscale at selected PLDs demonstrate good vessel visualization and show the dynamic passage of the bolus through the vasculature. Some loss of signal is noted where the blood is changing direction rapidly (red arrows) and near the frontal sinuses, where there is considerable B_0_ inhomogeneity (purple arrows). (B) Example sagittal and transverse slices of the perfusion images reconstructed from the same raw dataset, showing mainly macrovascular signal at early PLDs but perfusion of the tissue at later PLDs. However, SNR at later timepoints is low, limiting the ability to visualize tissue perfusion clearly (orange arrow). PLDs are indicated above each subfigure

Example 4D CAPRIA data acquired with a CFA schedule and reconstructed with SENSE are shown in Figure 3. Despite the high undersampling factor (*R* = 19) the reconstructed angiograms clearly show the flow of blood through the arterial system. Note that, due to the long labeling duration (1.4 s), the first frame shows much of the vasculature filled with labeled blood, but inflow visualization can also be achieved retrospectively (see the Discussion section). There was some signal loss in proximal vessels, most likely due to flow‐induced dephasing where the blood is changing direction rapidly, or due to the presence of nearby air‐tissue interfaces, resulting in dephasing due to strong B_0_ inhomogeneity. Reduced signal intensity at later timepoints is also evident due to the attenuating effect of the CFA excitation pulses.

Within the perfusion images reconstructed from the same raw k‐space data, blood was well visualized at early timepoints, showing ASL signal within the large vessels and in tissue regions where blood arrives quickly (e.g., some deep gray matter structures). However, the majority of the tissue is not perfused until later timepoints when the attenuation effect has become more significant. This effect, combined with the more dispersed blood signal and greater T1 decay, causes the perfusion image SNR to be very low.

**FIGURE 4:**
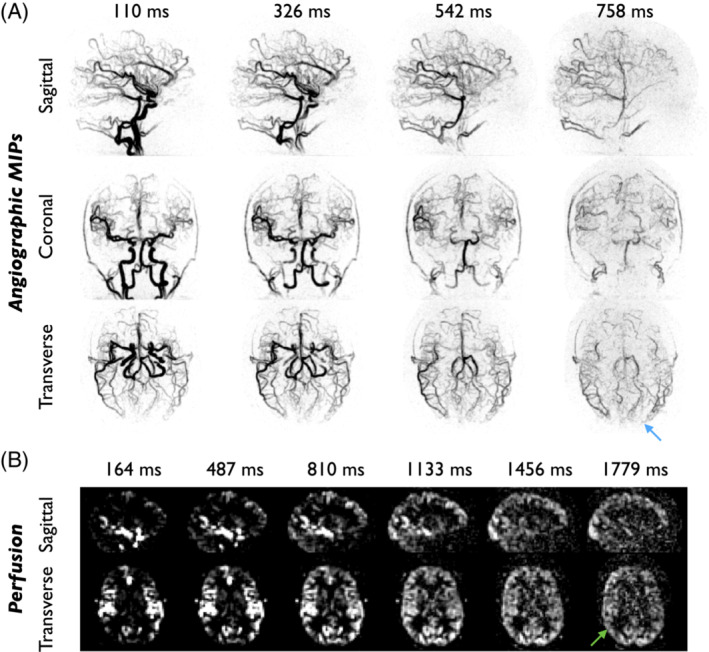
Example CAPRIA SENSE data from the same subject shown in Figure 3 (subject 1), but this time acquired with a variable flip angle (VFA) scheme. (A) Despite the considerably lower initial flip angle, angiographic maximum intensity projections at early PLDs show comparable image quality to the CFA data, whereas the visualization of distal vessels at later PLDs is improved (blue arrow). (B) Perfusion images reconstructed from the same raw dataset demonstrate much stronger perfusion signal at later PLDs than the CFA data (green arrow)

Figure 4 shows angiographic and perfusion SENSE reconstructions from the same subject as Figure 3, but this time acquired with a VFA schedule. Despite the low initial flip angle (2°), the angiographic images at early timepoints are qualitatively similar to those produced by the CFA approach. The signal does not reduce over time and visualization of distal blood vessels at later timepoints is improved. As predicted from simulations, the increase in signal at later timepoints achieved with the VFA approach means tissue perfusion is much more clearly visualized. In addition, the spoiled gradient echo 3D radial readout results in isotropic spatial resolution with no through‐slice blurring, distortion, or dropout artifacts, which are common in other ASL perfusion imaging approaches.^10^

**FIGURE 5:**
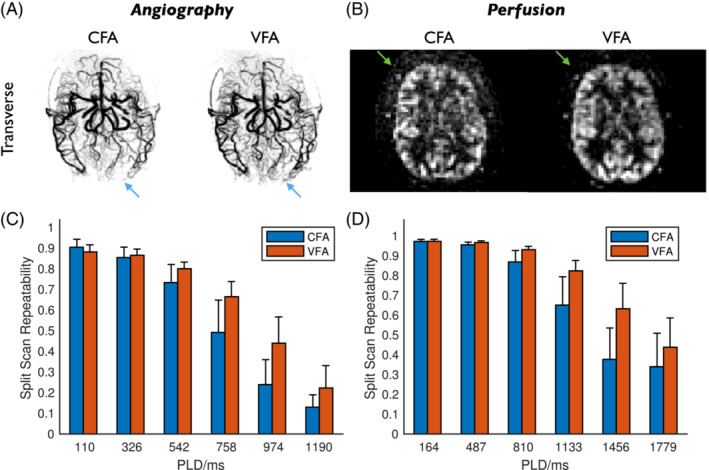
Comparison of CFA and VFA protocols. (A) Temporal mean angiographic transverse maximum intensity projections of subject 1, showing comparable image quality, with slightly better distal vessel visibility in the VFA protocol (blue arrows); (B) Temporal mean of transverse perfusion images with PLDs >1 s in subject 1, showing reduced noise in the VFA data (green arrows). (C) Split scan repeatability (correlation coefficients) for angiography, showing the mean and standard deviation across subjects at each of the first six PLDs. (D) Split scan repeatability for perfusion imaging at all PLDs. CFA and VFA images in (A) and (B) are scaled separately for improved visualization. CFA and VFA differences are significant (*p* < 0.001) for both angiography and perfusion imaging

The benefit of VFA over CFA is highlighted in Figure 5. Temporal mean images demonstrate the improved distal vessel visibility for angiography and the higher apparent SNR for perfusion imaging. The split‐scan repeatability metrics quantitatively demonstrate an improvement in signal stability for VFA compared with CFA, particularly at later PLDs, which was significant (*p* < 0.001) for both angiography and perfusion imaging: an increase in repeatability of up to 84% was found for angiography (at PLD = 974 ms) and up to 68% for perfusion imaging (at PLD = 1456 ms).

### 3.3 Comparison of reconstruction approaches

**FIGURE 6:**
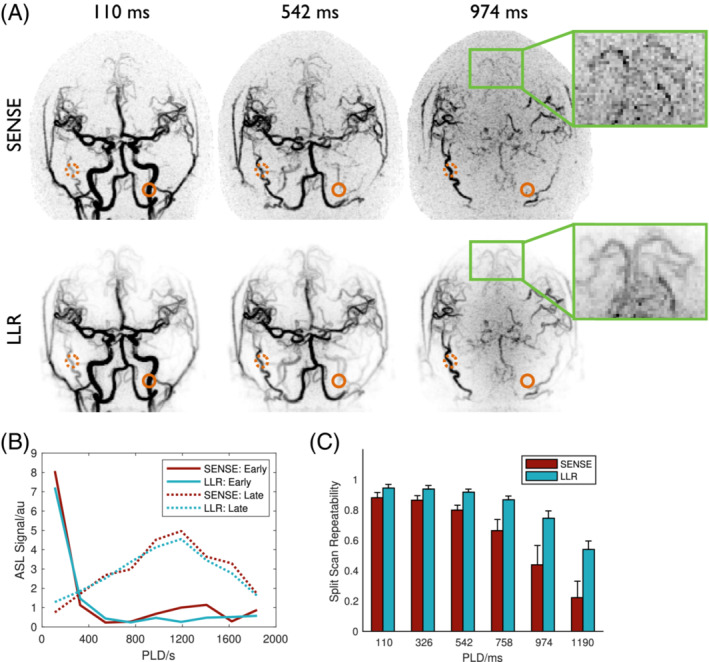
Comparison of angiographic image reconstruction approaches applied to VFA data. (A) Coronal MIPs of selected frames from SENSE (top row) and LLR (bottom row) reconstructions in subject 2, with the inset showing a zoomed and re‐windowed region highlighting the denoising effect of the LLR reconstruction that improves distal vessel visibility. (B) Signal timeseries from example voxels with early (solid lines) and late (dotted lines) blood arrival (highlighted with circles in A), demonstrating the minimal temporal bias introduced by using the LLR reconstruction approach. (C) Mean and standard deviation split scan repeatability across all subjects for the first six PLDs showing the significant (*p* < 0.001) improvement in signal stability achievable using the LLR method, particularly at later time points

A comparison of SENSE and LLR approaches for VFA angiographic reconstructions is shown in Figure 6. Although SENSE gives a reasonably clear visualization of the vessels at early timepoints, there is some residual background noise which increases over time, likely due to increased physiological noise from the higher static tissue signal associated with the VFA schedule, obscuring some smaller distal vessels (Figure 6A, inset). In contrast, the LLR approach reduces a lot of this background noise, giving a much clearer depiction of the arterial system, especially the distal vessels at later timepoints.

One potential concern with reconstruction approaches such as LLR is that overregularization could bias the signal time courses. Figure 6B shows the angiographic signal in two example voxels with early and late blood arrival, demonstrating that the LLR approach has a denoising effect but does not strongly distort the signal evolution.

LLR also significantly improves the split scan repeatability metric (Figure 6C, *p* < 0.001), implying improved signal stability, especially at later PLDs. An increase in repeatability of up to 143% relative to SENSE was observed (at PLD = 1190 ms).

**FIGURE 7:**
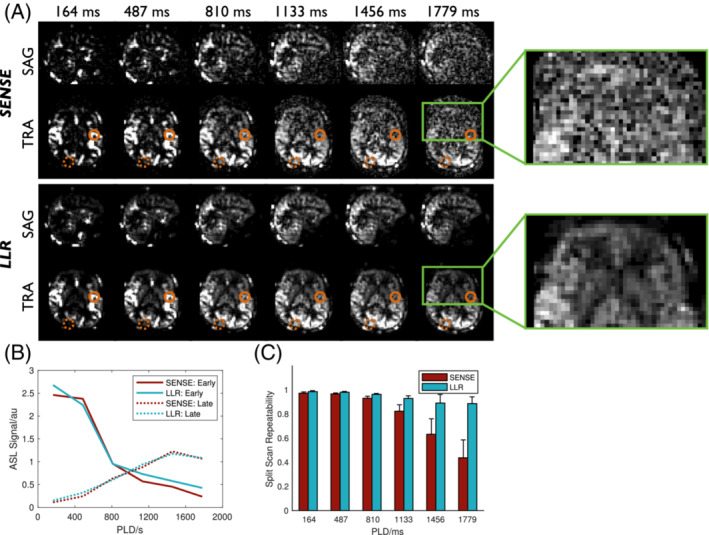
Comparison of perfusion image reconstruction approaches applied to VFA data. (A) Sagittal (SAG) and transverse (TRA) slices at all PLDs from SENSE (top row) and LLR (bottom row) reconstructions in subject 3, with the inset showing a zoomed and re‐windowed region highlighting the noise reduction achieved with the LLR reconstruction. (B) Signal timeseries from example voxels with early (solid lines) and late (dotted lines) blood arrival (highlighted with circles in A), demonstrating the minimal temporal bias introduced by using the LLR reconstruction approach. (C) Mean and standard deviation split scan reproducibility across all subjects for all PLDs showing the significant (*p* < 0.001) improvement in signal stability achievable using the LLR method, particularly at later time points

Figure 7 compares SENSE and LLR for VFA perfusion imaging. The improvement in image quality achievable with LLR is even more apparent in this case, particularly the denoising effect on the later PLD images when the signal is mostly perfusion weighted (with minimal macrovascular signal). As before, the LLR reconstruction does not appear to strongly bias the signal timecourses (Figure 7B) and gives a significant (*p* < 0.001) improvement in split scan repeatability (up to 103% at PLD = 1779 ms).

LLR angiographic and perfusion reconstructions across multiple timepoints for all subjects can be seen in Videos S1 and S2, respectively, demonstrating the consistent image quality that was obtained in all cases.

### 3.4 Comparison with conventional methods

**FIGURE 8:**
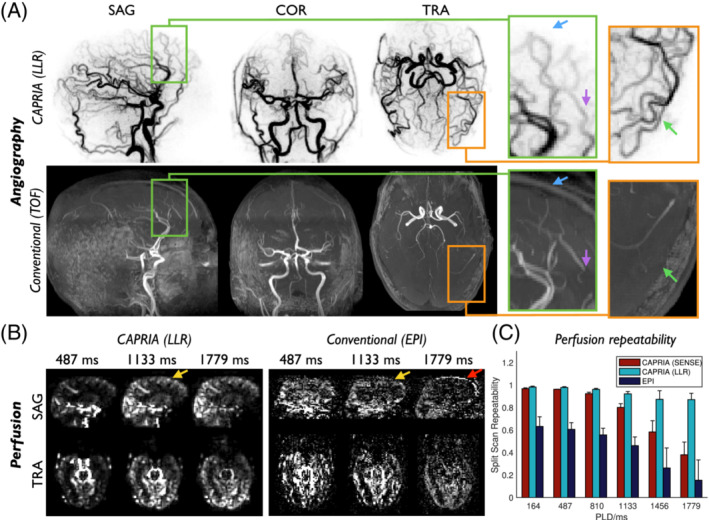
Comparison of CAPRIA with time‐matched conventional angiography (TOF) and perfusion imaging (multi‐PLD PCASL with an EPI readout). (A) The temporal MIP of the first four frames from the CAPRIA angiographic LLR reconstruction in subject 2 is shown alongside a conventional TOF acquisition, using spatial MIPs in all three directions. Although the higher in‐plane resolution of the TOF data allows finer vessel structure to be seen, the majority of the distal vessels are obscured by overlying static tissue signal, which is not the case for CAPRIA (see orange zoomed section, green arrows). In addition, TOF suffers from venous contamination (blue arrow) and slab boundary artifacts (purple arrow), as shown in the green zoomed section. (B) Example sagittal and transverse slices at three PLDs from the CAPRIA and conventional perfusion images acquired in the same subject. Similar perfusion patterns can be observed, although the multi‐slice EPI readout results in much longer effective PLDs in superior slices, impeding the visualization of the perfusion signal (yellow arrows). The EPI data is also visibly much noisier and some motion artifact can be seen (red arrow). (C) Split scan repeatability of the perfusion data, averaged across the 3 subjects where EPI data were available, is significantly (*p* < 0.001) higher for CAPRIA than conventional EPI, for both SENSE and LLR reconstructions. Note that the EPI repeatability values have been corrected for the reduced data used to calculate this metric (see the Methods section)

Figure 8 shows a comparison of CAPRIA angiograms and perfusion maps (reconstructed using LLR) with total‐time‐matched conventional TOF angiograms and multi‐PLD PCASL EPI perfusion images. Similar features in the large vessels were observed in angiograms derived from both techniques, although the slightly higher in‐plane resolution of the TOF angiograms gives slightly better vessel definition. However, it is apparent that CAPRIA has a number of advantages over the TOF protocol used in this study: (1) the distal vessels are much better visualized in maximum intensity projections due to the removal of all overlying static tissue signal by subtraction; (2) it does not suffer from slab boundary artifacts; (3) no venous signal is present, as is apparent in the TOF data, particularly in the most superior slab; and (4) it provides dynamic, rather than static, information about blood flow through the arteries. Similar features were observed in both modalities across all subjects.

CAPRIA also demonstrated advantages over conventional multi‐PLD PCASL perfusion imaging (Figure 8B): as with other 3D readouts, all CAPRIA voxels within a given time point have the same effective PLD, whereas with a 2D multi‐slice EPI readout, the effective PLD increases considerably in more superior slices. This is particularly apparent in the protocol used in this study, since a large number of thin slices were acquired to match the isotropic resolution of the CAPRIA readout, causing the EPI readout to take ˜1.7 s, which impeded the visualization of perfusion in superior brain regions. In an inferior slice where the PLDs are relatively well matched, similar patterns of blood flow are observed, although the EPI data are visibly noisier. This was reflected in the split‐scan repeatability metrics (Figure 8C), which showed significantly lower repeatability for EPI than for CAPRIA, whether a SENSE or LLR reconstruction was used (*p* < 0.001).

### 3.5 Potential for scan time reduction

**FIGURE 9:**
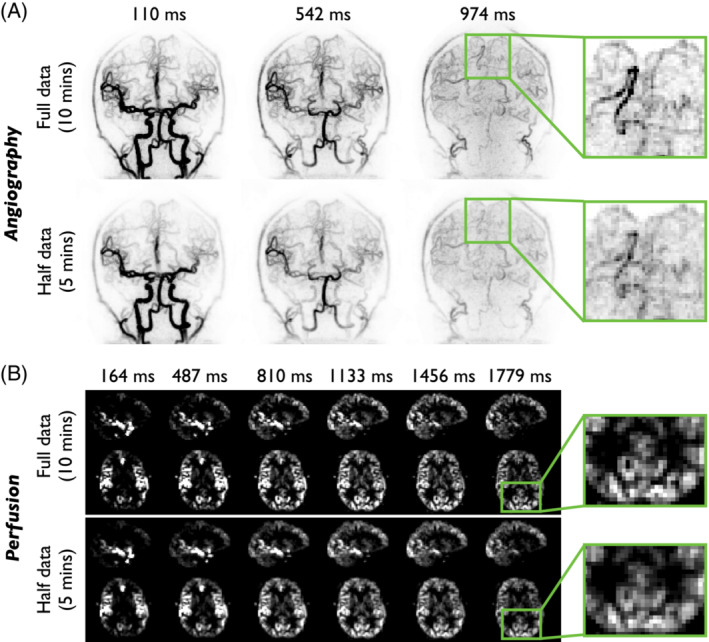
Potential for scan time reduction: Example CAPRIA angiographic coronal MIPs (A) and sagittal/transverse perfusion slices (B) from subject 1 at a range of PLDs. LLR reconstructions using the full data set (10 min) and only the first half of the data (5 min) are shown. The majority of image features are retained when only half the data are used, although a slight loss of distal vessel visibility and a small increase in blurring are apparent (see zoomed and re‐windowed insets)

Examples of CAPRIA angiograms and perfusion images reconstructed using LLR from the first 5 min of data only (identical to a prospective shorter scan) are compared with those reconstructed from the full 10 min of data in Figure 9. Despite the undersampling factors being doubled there is relatively little difference between the two cases, with all the major features of both the angiograms and perfusion images still clearly visible. However, the zoomed insets do demonstrate a slight loss of spatial resolution and fine vessel detail, as might be expected due to the reduced sampling at higher spatial frequencies.

## 4 DISCUSSION

In this study we have demonstrated the extension of CAPRIA to a 4D technique, allowing time‐resolved 3D angiograms and perfusion images to be obtained from a single non‐contrast acquisition. We further optimized the image quality using a VFA readout scheme to reduce the signal attenuation at early timepoints and boost the weaker signal at later timepoints. We also leveraged the spatiotemporal correlations in the data using an LLR reconstruction method to significantly reduce the noise level, giving a much clearer delineation of distal vessels and the weaker perfusion signal at long PLDs. 4D CAPRIA was shown to compare favorably against conventional time‐matched angiographic and perfusion imaging methods, improving distal vessel visibility and perfusion signal stability. The potential to reduce the acquisition to a more clinically feasible 5 min scan was also demonstrated.

### 4.1 Pulse sequence design

The extension of the original golden ratio approach^12^ to allow sampling in 3D k‐space^22^ was ideal for 4D CAPRIA, enabling flexibility in the temporal and spatial resolution of the reconstruction to allow either angiographic or perfusion‐like images to be reconstructed at any PLD within the readout window. Despite the high undersampling factors (*R* = 19), the sparse and high SNR nature of the angiographic signal allowed reconstruction with minimal apparent artifacts due to the incoherent aliasing that arises from this trajectory, as previously noted for similar acquisition schemes.^23^, ^24^, ^42^ The flexibility to retrospectively choose the spatiotemporal resolution allowed perfusion images to be reconstructed with acceleration factors close to one, which was important for this much lower SNR and less sparse signal.

In this study, only a single temporal resolution was demonstrated for angiography and perfusion imaging reconstructions, although any temporal window up to a maximum value, *t*_max_, could be chosen, as was demonstrated previously.^13^ The *t*_max_ approach has the advantage that data can be retrospectively divided into different sections acquired during different time periods (e.g., the first and second half of the scan), which are exactly equivalent to performing shorter scans with higher acceleration factors. This feature could be useful for discarding data acquired during severe motion, for example. However, for temporal windows much smaller or larger than *t*_max_ the trajectory will stray further from the near‐ideal golden ratio ordering, resulting in reduced SNR efficiency and worse undersampling artifacts. An alternative approach that maintains exact golden ratio ordering for any temporal window^43^ has shown promise for application in combined angiography and perfusion imaging.^14^ This method could be easily adapted for use with the 3D radial trajectory used in this study and will be explored in future work.

The use of a relatively long labeling duration (1.4 s) meant that the dynamic angiograms mostly show the outflow of labeled blood water, which might be less intuitive to interpret than the inflow of labeled blood that can be visualized with pulsed ASL techniques.^43^, ^44^ However, inflow can be visualized from this kind of data using an inflow subtraction technique,^45^, ^46^, ^47^ as shown in Video S3. However, this reduces SNR and does not easily account for voxels with delayed blood arrival (*δ*_*t*_ *> τ*) or for the potential increase in signal over time resulting from the use of a VFA schedule (see Figure 2B). An alternative approach is to fit a kinetic model and then simulate images at arbitrary timepoints that would be produced with an infinitely long labeling duration, with the effects of RF attenuation and T1 decay removed.^26^

### 4.2 Flip angle schedules

The quadratic VFA scheme used in this study gives an additional degree of freedom in the pulse sequence design, allowing reduced signal attenuation at early timepoints while boosting the ASL signal at later timepoints. Simulations and in vivo experiments both confirm that this approach is highly beneficial to boost the lower SNR perfusion signal, particularly because of its later arrival in the tissue. Although the angiographic signal is considerably reduced when using small initial flip angles, this did not seem to cause an obvious loss in image quality. This suggests the angiographic signal already has a sufficiently high SNR and the apparent “noise” in these images is dominated by aliased signal and physiological noise (which both scale with signal strength) rather than thermal noise. In contrast, the perfusion signal, which is much weaker due to blood dispersal and the greater degree of T1 decay by the time the labeled water exchanges into tissue, clearly benefits from the increase in signal achieved with the VFA scheme at later timepoints. However, it should be noted that the specific VFA optimization performed here was relevant for the blood arrival times of up to 1 s within the vessels and up to 2 s to reach the tissue and should be recalculated for patient cohorts where very delayed blood arrival is expected.

An arbitrary weighting factor of ½ was chosen to upweight the perfusion contribution in the flip angle optimization. Other factors could also be chosen, based on the relative importance of angiographic and perfusion information for any given application. Although we chose to focus on CFA and quadratic^25^ VFA schedules in this work, other VFA schemes could also be explored, such as linear variations^48^ or the use of a recursive formula to maintain the ASL signal at a constant level.^7^, ^49^, ^50^ The use of a balanced steady‐state free precession readout could lead to a significant increase in SNR efficiency^44^ and might also benefit from a VFA schedule, although a separate optimization would need to be performed to account for the refocusing of transverse magnetization. However, this approach can suffer from significant signal loss when using a large field of view (FOV, e.g., the whole head) at 3T due to B_0_ inhomogeneity.^45^

### 4.3 Reconstruction approaches

The LLR reconstruction method was found to give a significant improvement in image quality for both angiography and perfusion imaging compared with conventional SENSE. This approach exploits the very strong correlations between the signal timecourses across voxels within any given patch of the image (e.g., they typically have similar arrival times and signal dispersion), meaning that the signal can be well represented by a low rank model. Therefore, enforcing an LLR solution that is consistent with the measured data suppresses noise and residual aliasing very effectively. The variation in the k‐space trajectory across timepoints combined with a spatiotemporal regularized reconstruction like LLR can also be thought of as reducing the effective undersampling factor of the reconstruction by sharing information across time, thereby reducing noise amplification, which has been shown to benefit similar techniques in ASL perfusion imaging^51^ and angiography.^34^ However, it is clear that such regularized approaches have the potential to bias the signal, both spatially and temporally, which could have implications for image interpretation and kinetic model fitting. The regularization factors used here were chosen to minimize this issue, although the optimum choice may be application dependent, and the interaction with model fitting needs to be explored in future work.

Although the image reconstruction comparison in this study focused on VFA data, a similar benefit is likely to be gained in using a LLR reconstruction on CFA data also. In our preliminary experiments, we found LLR to provide more robust results than other compressed‐sensing reconstruction approaches, so we focused on that technique here. However, other techniques have been applied to ASL angiography and perfusion imaging, such as wavelets/total variation,^52^ magnitude subtraction sparsity,^53^ spatial sparsity with temporal smoothness,^34^ total generalized variation,^51^ and low rank + sparse with non‐local filters.^54^ A comprehensive comparison is beyond the scope of the current work but would be interesting to explore in the future.

### 4.4 Comparison with conventional methods

The angiograms and perfusion images obtained from a single 4D CAPRIA scan were shown to have considerable advantages over separately acquired time‐matched data generated by conventional techniques. The complete removal of static tissue through subtraction greatly facilitates the visualization of small distal vessels in maximum intensity projections compared with TOF, and the additional time‐resolved information available could provide additional insights into hindered blood flow through the vascular system or allow separation of the arterial and venous phases of flow through arteriovenous malformations, for example. For perfusion imaging, image quality was shown to be considerably higher for 4D CAPRIA than multi‐PLD PCASL with a 2D multi‐slice EPI readout, and 4D CAPRIA also benefits from minimal distortion and signal dropout artifacts.

It might be expected that the reduced flip angles used for CAPRIA, relative to conventional angiographic or perfusion imaging methods, as well as the use of only the central portion of k‐space for perfusion imaging, might reduce its SNR efficiency. However, as discussed in previous work,^13^ this is counterbalanced by: (a) the greatly increased time spent sampling the ASL signal across the long readout period; and (b) the longer scan time that can be spent acquiring signal that contributes to the SNR of both angiographic and perfusion images, compared to acquiring them separately. In addition, the regular sampling of the center of k‐space likely contributes to improved signal stability and reduced sensitivity to physiological noise.

However, due to the requirement to match scan times and limitations of the pulse sequences used, it was not possible to exactly match the conventional protocols to the CAPRIA acquisition in certain respects. In particular, the use of a multi‐slice 2D EPI readout for perfusion imaging meant that the effective PLDs were considerably longer than those of the CAPRIA data in superior slices, which contributed to the poorer perfusion image quality observed. Although this is a real disadvantage of 2D multi‐slice methods, a comparison to a 3D readout such as 3D‐GRASE,^55^ where the PLDs could be closely matched and the SNR advantage of using a 3D readout is comparable between the two methods, would be desirable. In addition, the TOF protocol was not specifically optimized for visualizing small distal vessels, so it might have performed better if this were the case. In particular, at high spatial resolution (e.g., 0.5 mm isotropic) but with limited spatial coverage, TOF angiography could become even more competitive. The extent to which CAPRIA angiographic spatial resolution can be improved to match this kind of acquisition without compromising perfusion image quality is not yet known. Such investigations will be explored in future work.

### 4.5 Comparison to related work

Other approaches for combined angiography and perfusion imaging with ASL have also been proposed. One used time‐encoded^56^ PCASL in combination with two readout modules, which were separately optimized for angiographic and perfusion information.^11^ Another combined time‐encoded PCASL with a 2D multi‐slice golden ratio readout,^14^ which was utilized to vary both the temporal and spatial resolution of the reconstructions. The use of time‐encoding allows the generation of images at different effective delay times after the labeling period, with short delays used for angiography and longer delays for perfusion imaging, while minimizing the number of excitation pulses required after each ASL preparation. This reduces the attenuation of the ASL signal compared to the proposed approach, where temporal information is obtained only from the use of a long readout, and was shown to significantly increase SNR, especially for the perfusion phase.

However, the use of time‐encoding required the effective bolus duration to be short for angiography, which could reduce angiographic SNR. In addition, the relative timings of the images are linked to the PLD associated with each Hadamard encoding block, providing less flexibility than the continuous golden ratio approach used in this study. Finally, the same k‐space spokes had to be acquired many times to accommodate all the Hadamard encoding steps, which increased the undersampling factor relative to conventional PCASL, which would be particularly problematic for a 3D radial trajectory like the one proposed in this study.

Compared to multi‐slice 2D methods, a 3D golden ratio readout has the additional advantages of SNR efficiency and the ability to reconstruct signals in all voxels across all timepoints. Another option is a 3D stack‐of‐stars radial trajectory,^43^, ^53^ which uses in‐plane radial and through‐plane Cartesian sampling. This approach may have some advantages when a limited number of slices are required, although the ability to undersample in the through‐slice direction is perhaps more limited. However, the optimal combination of time‐encoding and readout approach is not yet clear and may be somewhat application dependent, so will be the subject of future investigations.

### 4.6 Limitations

This study had a number of limitations which could be improved upon in future work. Firstly, the use of non‐selective excitation means that the labeling plane is included in the imaging FOV. The different saturation effects on the static tissue at the labeling plane in label and control conditions creates a large difference signal which aliases into the brain, degrading image quality. A very large reconstructed FOV is also necessary to encompass the sensitive area of the receive coil, which increases the computational burden of the reconstruction. In addition, depending on the efficiency of the RF transmit coil inferior to the neck and the rate at which blood is replenished in proximal vessels, inflowing blood could be partially saturated by the excitation pulses before it is labeled by a subsequent ASL preparation, reducing the measured signal. A slab‐selective excitation is therefore likely to be beneficial. Methods to minimize dephasing in proximal vessels due to flow or B_0_ effects, such as flow‐compensation or a reduced TE,^23^, ^57^ would also be desirable. TE reduction could be achieved through more aggressive readout partial Fourier, at a cost of reduced k‐space sampling, or increased readout bandwidth, which would reduce SNR.

Secondly, the magnetization preparation could be improved: only a pre‐saturation module was used for background suppression. Additional inversion pulses could reduce physiological noise and improve image quality.^58^ The PCASL pulse train duration could also be extended to improve signal strength, but must be balanced against the cost of increased imaging time or undersampling factor. The scan time of 10 min used for the majority of this study may already be too long to fit into a busy clinical protocol, although the relatively small change in image quality when reducing this to a 5 min scan, as shown in Figure 9, suggests 4D CAPRIA could be feasible for future clinical applications.

Thirdly, we aimed here to improve qualitative angiography and perfusion imaging, but the extraction of quantitative physiological parameters would be useful. Adaptations to the physiological models described here to include dispersion would be necessary for this, as well as procedures for signal calibration, and will be investigated in the near future.

Finally, validation of CAPRIA in a larger number of healthy volunteers and in patients with a range of cerebrovascular diseases, including comparisons to conventional angiographic and perfusion imaging methods, is necessary to more robustly establish its utility, particularly under pathological flow conditions, and robustness to patient motion.

## 5 CONCLUSIONS

4D CAPRIA provides time‐resolved angiographic and perfusion information from a single scan across the whole head, with minimal blurring, distortion, or dropout artifacts. A quadratic VFA scheme greatly improves image quality at later timepoints, especially for perfusion imaging, and an LLR reconstruction scheme makes further improvements to noise reduction, allowing the clear depiction of distal vessels and late tissue perfusion. 4D CAPRIA showed good performance relative to time‐matched TOF angiography and multi‐PLD PCASL perfusion imaging, including higher perfusion image repeatability.

## CONFLICT OF INTEREST

TO is the author of a US patent relating to the CAPRIA technique described in this manuscript.

## Supporting information


**Figure S1:** An example vessel mask (white) and dilated vessel mask (red) used for angiography repeatability analysis, shown as a single transverse slice (A) and a transverse maximum intensity projection (B).

**Video S1:** The first six frames of LLR angiographic reconstructions for all subjects (columns), with MIPs in sagittal (top row), coronal (middle row) and transverse (bottom row) views.

**Video S2:** All frames of LLR perfusion reconstructions for all subjects (columns), with example sagittal (top row), coronal (middle row) and transverse (bottom row) slices shown.

**Video S3:** The first six frames of LLR angiographic reconstructions for all subjects (columns), with MIPs in sagittal (top row), coronal (middle row) and transverse (bottom row) views, after inflow subtraction has been applied.

## Data Availability

CAPRIA image reconstruction and signal simulation code used in this study are openly available in GitHub (https://github.com/tomokell/capria_tools) and Zenodo (http://doi.org/10.5281/zenodo.6821643).^59^ Data underlying the plots and code to produce them, including statistical analysis, are openly available in Zenodo (http://doi.org/10.5281/zenodo.7390441).^60^ Deidentified image data will be made available on the WIN Open Data server. This is currently in development. Register here to find out when materials are available for download: https://web.maillist.ox.ac.uk/ox/subscribe/win‐open‐data.
