# Supplementary material for: Optimization of 4D combined angiography and perfusion using radial imaging and arterial spin labeling
Source: Magn Reson Med. 2022 Dec 19;89(5):1853–70. doi: 10.1002/mrm.29558 (PMC10952652; doi:10.1002/mrm.29558)
Supplement: Supplementary file 1 — Figure S1: An example vessel mask (white) and dilated vessel mask (red) used for angiography repeatability analysis, shown as a single transverse slice (A) and a transverse maximum intensity projection (B). [file MRM-89-1853-s002.pdf]

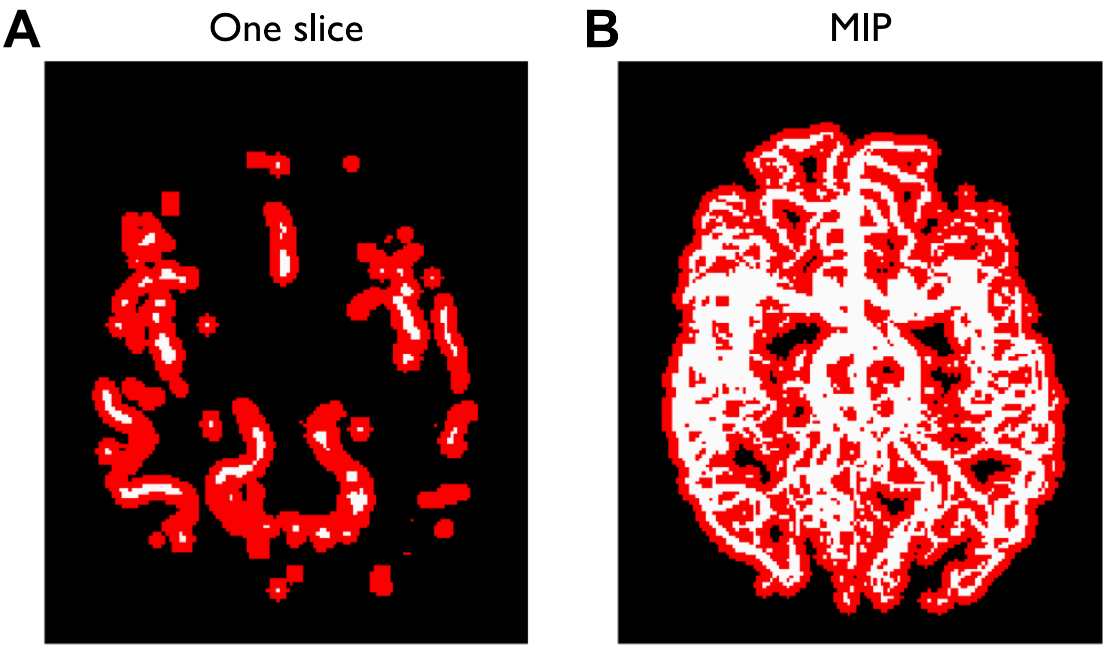


**Supporting Information Figure S1**: An example vessel mask (white) and dilated vessel mask (red) used for angiography repeatability analysis, shown as a single transverse slice (A) and a transverse maximum intensity projection (B).
